# A method of determining heated ancient nephrite jades in China

**DOI:** 10.1038/s41598-018-30564-w

**Published:** 2018-09-10

**Authors:** Yi Bao, Chaohong Zhao, Yuesheng Li, Xuemei Yun

**Affiliations:** 1 Departments of Materials Science, Fudan University, Shanghai, 200433 P. R. China; 2 School of Archaeology and Museology, Peking University, Beijing, 100871 P. R. China; 3 Research Center for the Development of Geosciences, China University of Geosciences (Beijing), Beijing, 100083 P. R. China

## Abstract

Ancient jade is one of the most significant cultural relics in China. Despite the increasingly important role of heated jades in ancient jade study, there are still few in-depth material studies on heated jades for the time being, which results in limitations on archaeological research and conservation. In order to evaluate the appearance change of nephrite jades, we employ the heating simulation experiment to present these change under different heating temperature ranging from 100 °C to 1300 °C. In this study, a nondestructive identification method proposed is to help determine whether the ancient jades were heated before and what the approximate heating temperature was from 100 °C to 1300 °C though phase transformation, structure change and colour variation with several advanced characterization methods such as the X-ray diffraction (XRD), the thermo-gravimetric analyser (TGA), the ultraviolet-visible spectrophotometer (UV-Vis), the Fourier transform infrared spectroscopy (FTIR) and the scanning electron microscope (SEM). The hierarchy of colour (from ~500 °C) and structure (from ~800 °C) on heated jades in the jade research field were first proposed and discuss here. This method is considered useful and valuable in the study and protection of Chinese ancient jades.

## Introduction

As one of the most characteristic relics in China, ancient jade is a significant representation of traditional Chinese culture. Throughout the world history, Chinese jade culture is the only one without ceasing for more than 8000 years^1^. Plenty of studies focus on ancient jade for the conservation and promotion of this cultural heritage.

China has a long history of fire tradition to burn oblations in ceremonies (Liao Ji)^2^. Jades are deemed to be one of the oblations in Chinese fire tradition^3^. The hearths of fire ceremonies were excavated in the 8000-year-old Hongshan Culture relic in Liaoning Province^4^, which is an important origin of Chinese jade culture^1^. Similar relics were also discovered in the Yinxu Site, a capital of the Shang Dynasty^5^, which is thousands years later than the Hongshan Culture. All these archaeological findings indicate that the fire ceremony has a long history in China. It was recently verified that the ancient jades found in the Yinxu Site were heated before (in press). It is also the first time that academics have found proof that jades were heated in ancient time. It is highly possible that a large number of burned jades were heated in the fire tradition in China and the practice lasted for thousands of years^6^. Therefore, it is necessary to carry out a systematic study in this practice.

It is important to understand the heating process of jade. Firstly, the result of the experiment can help us know more about the heated ancient jades. Secondly, the appearance variation observed from experiments can help researchers to determine whether the jades were heated before. Lastly, the result will lay a significant theoretical foundation for the conservation of heated-ancient-jades and will help us determine the best practice of protection in different on-site environment conditions.

The heating of jade was first pointed out by Wu^7^. Wu stated that an artifact was subjected to heating by either “anthropogenic fire” or “Dihuo”^8^. The heating of jades was considered as an important component of ancient ceremonies or a method to make the jade cutting easier. Allen *et al*.^9^ carried out the first jade heating experiments. Other researchers also conducted the simulation experiment subsequently^10–12^. However, former studies usually only focused on conditions below 800 °C. The relation between holding time and reaction has never been discussed before. Results from these experiments focused on low-iron samples and little discussed on change mechanism in depth. This paper will take both low-iron and high-iron nephrite samples for experiments and discuss more on these questions.

This paper shows the appearance change of nephrite jades under different temperature ranges and analyses its mechanism. Phase transformation, structure change, and colour variation are the three main topics we will discuss in this paper. This paper first proposes and discusses the hierarchy of structure and colour on heated jades in the jade research field. As a result, a reliable method is proposed to determine whether the ancient jades were heated before.

## Materials and Methods

### Materials

The 4 samples in this paper are all nephrite. Nephrite [Ca_2_(Mg, Fe)_5_Si_8_O_22_(OH)_2_] consists of two minerals of the amphibole group, tremolite and actinolite. These two minerals are distinguished by the ratio of magnesium and iron in the chemical composition. The mineral is tremolite when *f* = *Mg/*(*Mg* + *Fe)* ≥ 0.9; the mineral is actinolite when *f* is 0.5–0.9^13,14^. According to the level of *f*, the samples are divided into two different groups (Table 1). N1 and N2 are low-iron nephrite (tremolite), N3 and N4 are high-iron nephrite (actinolite).

**Table 1:** The characteristics of the nephrite jade samples.

		N-1	N-2	N-3	N-4
Colour		White with some brownish yellow variation	Greenish white	Green with much-dotted sap green	Sap green
Transparency		Semitranslucent	Semitranslucent	Semitranslucent	Semitranslucent
Gloss		Greasy lustre	Greasy lustre	Greasy lustre	Greasy lustre
Structure		Compact, crevices, some brownish yellow impurities	Even, compact, some waterline	Compact, big crystal, some dotted sap green impurities	Even, compact, fine
Atomic Concentration	Mg	13.3	11.0	11.5	11.5
	Fe	0.4	0.7	3.3	4.5
*Mg/(Mg* + *Fe)*		0.97	0.94	0.77	0.72
Mineral species		Tremolite	Tremolite	Actinolite	Actinolite

The iron and magnesium content of each sample was measured by the energy dispersive spectroscopy(EDS).

Different characteristics of the four samples are listed in Table 1. N-1 has many crevices. Its colour is white with some brownish-yellow variation. N-2 is greenish-white with even, compact structure and some “Shuixian” (the nervation structure with higher transparency). N-3 is green with many dotted sap green impurities and has many big crystals inside. N-4 has sap green colour and fine and even structure. These four samples are all semitranslucent. The tremolite and actinolite in these samples are all woven fibre structure.

**Figure 1:**
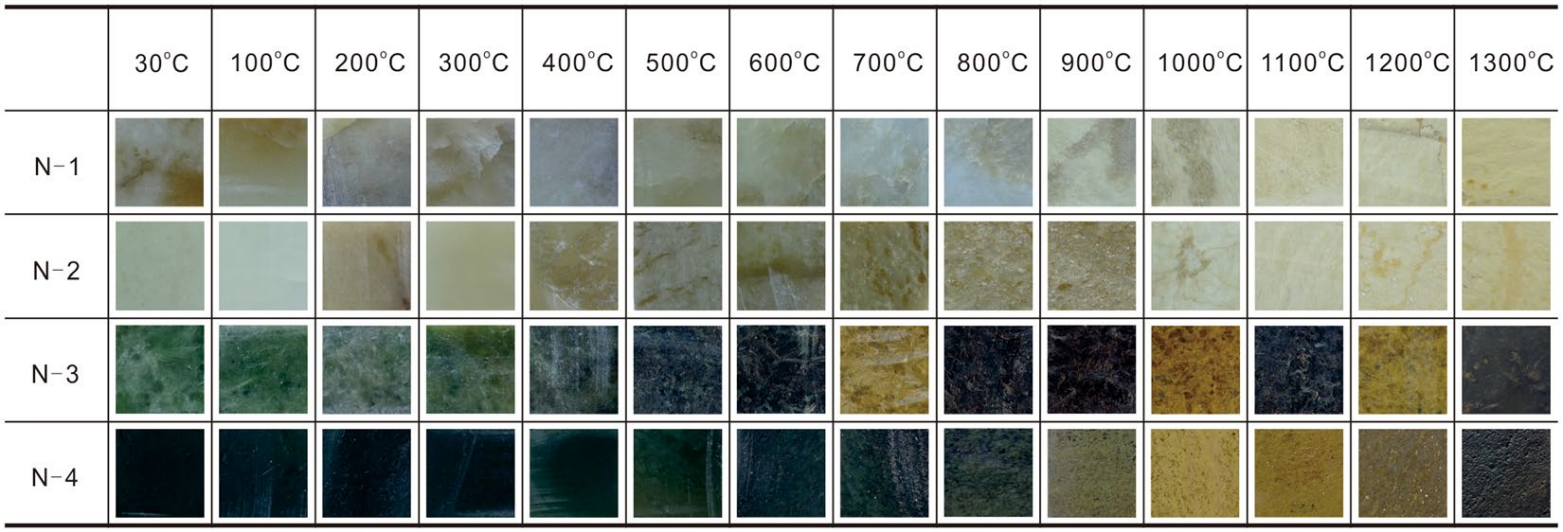
Photos of the heated nephrite jade samples.

Samples were cut with a water-cooled saw into square pieces (18 mm × 18 mm × 2 mm). These samples were all polished by SiC abrasive paper to make the lustre of the samples close to that of unearthed jades and to leave some polishing scratches. The different heating temperatures were appended to the names of the samples. For example, N-1-900 means that the N-1 was heated to 900 °C. The photos of polished samples are shown in Fig. 1.

### Heating Methods

In this study, muffle furnace (SGM 60/12 H, GSL 1400X) was used to heat the samples to a temperature between 100 °C and 1300 °C. Note that the holding time of previous studies ranges from 1 to 24 hours^9,15–18^. After a series of experiments, we found that longer holding time makes no remarkable difference to the results. At the same time, we found that the heating rate has little impact on the results as well. Thus we set the holding time to 30 minutes and the heating rate to 10 °C/min. Since the melting point of diopside is 1391 °C, the highest experiment temperature was set to 1300 °C. Experiments were performed with 100 °C increments from 100 °C to 1300 °C.

According to the level of technology development of thousands of years ago, heating the jade was possible. The flame temperature of burning wood is in 1000–1170 °C while the flame temperature is up to 1700 °C for the coal^19^. The people living thousands of years ago could proficiently make fire by wood^20^. The use of coal may started from Neolithic Age (5000 years B.P.) and is confirmed to exist in the Spring and Autumn Period (770-467 B.C.)^21^. Hence, 1300 °C is considered reasonable as the highest heating temperature.

## Results and Discussion

The results clearly show that the colour, transparency and lustre of the nephrite samples all changed (Fig. 1). These changes will be explained in three aspects phase transformation, structure change and colour change.

### Phase transformation

Tremolite-actinolite transforms into two kinds of pyroxenes, SiO_2_ and vaporous H_2_O after heating. Formula (1) exhibits this reaction:

1 $$\begin{array}{rcl}2C{a}_{2}{(Mg,Fe)}_{5}S{i}_{8}{O}_{22}{(OH)}_{2} & = & 4Ca(Mg,\,Fe)S{i}_{2}{O}_{6}+3{(Mg,Fe)}_{2}S{i}_{2}{O}_{6}+2Si{O}_{2}+2{H}_{2}O\\ \,\,Tremolite \mbox{-} Actinolite &  & \,Pyroxene\,1\,\,\,\,\,Pyroxene2\end{array}$$

**Figure 2:**
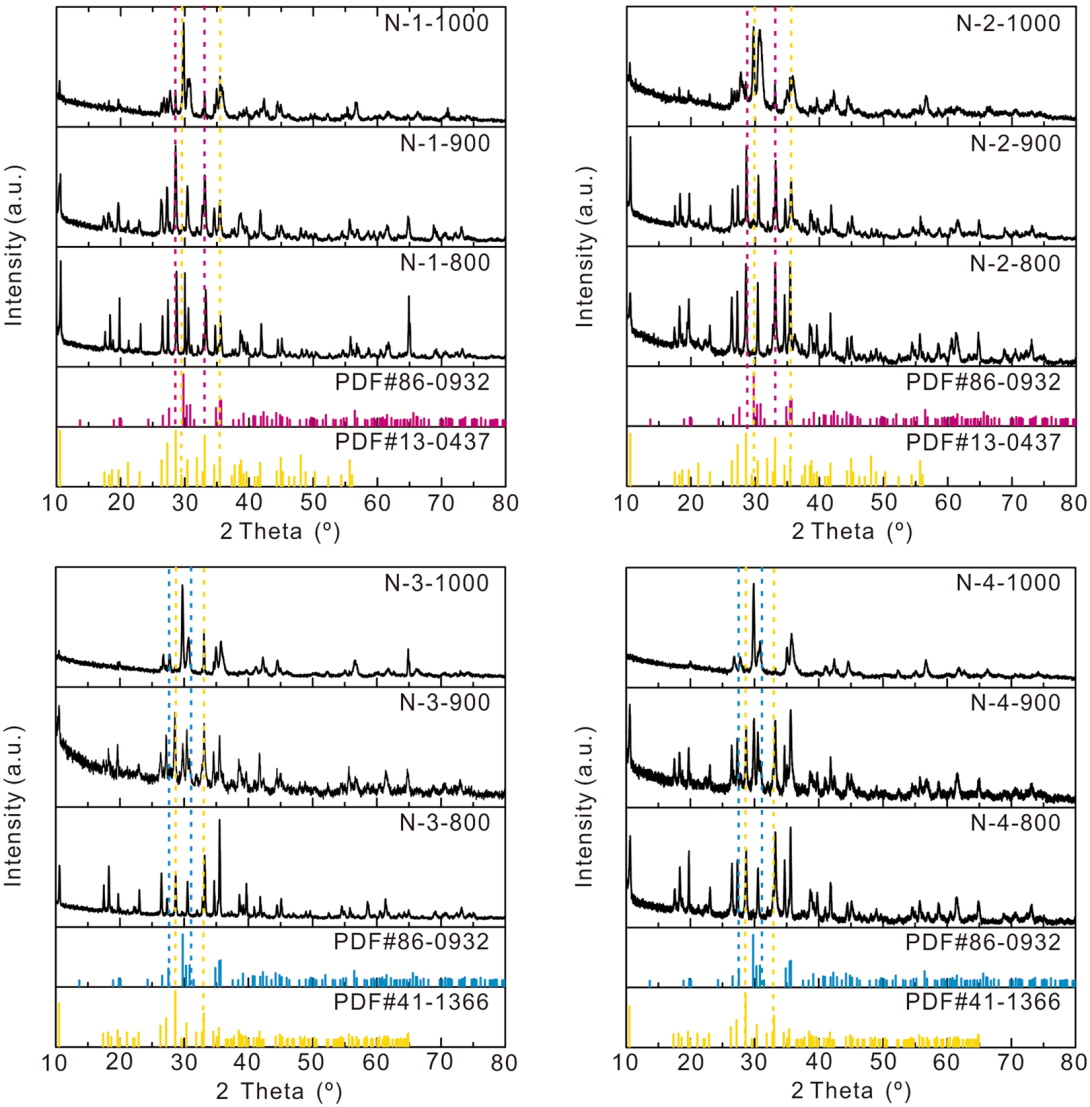
XRD data of the headed nephrite jade samples. PDF#86-0932, PDF#13-0437, PDF#41-1366 are representative XRD patterns of diopside, tremolite and actinolite, respectively. Tremolite-actinolite in N-1 and N-2 transform completely at ~1000 °C with good crystalline. Tremolite-actinolite in N-3 and N-4 transform partly at ~900 °C with low crystalline and transform completely at ~1000 °C with good crystalline.

These two kinds of pyroxenes belong to the diopside-hedenbergite series [Ca(Mg, Fe)Si_2_O_6_] and the enstatite-orthoferrosilite series [(Mg, Fe)_2_Si_2_O_6_]. The three silicate series in this formula are all Fe-Mg complete isomorphous series^13^. When *Fe* + *Mg* = *100%*, the iron content of the two new-formed pyroxenes should be 0–50% with high probability, since the iron content of tremolite-actinolite [Ca_2_(Mg, Fe)_5_Si_8_O_22_(OH)_2_] is 0–50%. Diopside and salite are the two end-member minerals in the diopside-hedenbergite series of which the iron content is between 0 and 50%. The three end-members with 0–50% iron content in the enstatite-orthoferrosilite series are enstatite, bronzite and hypersthene. In XRD data (Fig. 2), enstatite-hypersthene is the main mineral in four samples when heated over 1000 °C. This phenomenon relates to the big cellular molecular number of enstatite-hypersthene (Z = 16)^12,13^. Distinct XRD pattern of enstatite- hypersthene only exists when Z ≥ 16; although that value for diopside-salite is Z = 4^13^. Diopside-salite is the main transformational mineral phase in the XRD data (Fig. 2) of the samples heated below 1000 °C. The enstatite-hypersthene appears and corresponds to the theoretical ratio in the XRD data (Fig. 2) when the heating temperature is over 1000 °C.

The outcome SiO_2_ in this reaction has two type, crystalline state tridymite and amorphous state SiO_2_. There are several SiO_2_ minerals. It was suggested that cristobalite is one of the products during the heating process of quartz^15,22^. However, the formation temperature of cristobalite is over 1400 °C while the phase-transition temperature of nephrite is about 1000 °C. Therefore, the possibility of cristobalite is low. According to the formation rules of SiO_2_ as Formula (2)^13^, phase diagram of SiO_2_^13^ and the XRD data (Fig. 2), tridymite is the most probable product.

2 $$\begin{array}{c}\,\,\,\,573\,^\circ {\rm{C}}\,\,\,870\,^\circ {\rm{C}}\,\,\,1470\,^\circ {\rm{C}}\,\,\,1710\,^\circ {\rm{C}}\\ \alpha  \mbox{-} Quartz\to \beta  \mbox{-} Quartz\to Tridymite\to Cristobalite\to Quartz\,Glass\end{array}$$

Nevertheless, (ion) impurities influence the formation of the Si-O bond in the transform proceeding and lead to form amorphous SiO_2_ rather than crystal one. According to the XRD data (Fig. 2), the peaks of quartz are weak and the proportion of quartz is much lower than the theoretical value. And the proportion of tridymite improves little with the rise of heating temperature. As a result, the main type SiO_2_ is amorphous; and the less one is crystal tridymite.

**Figure 3:**
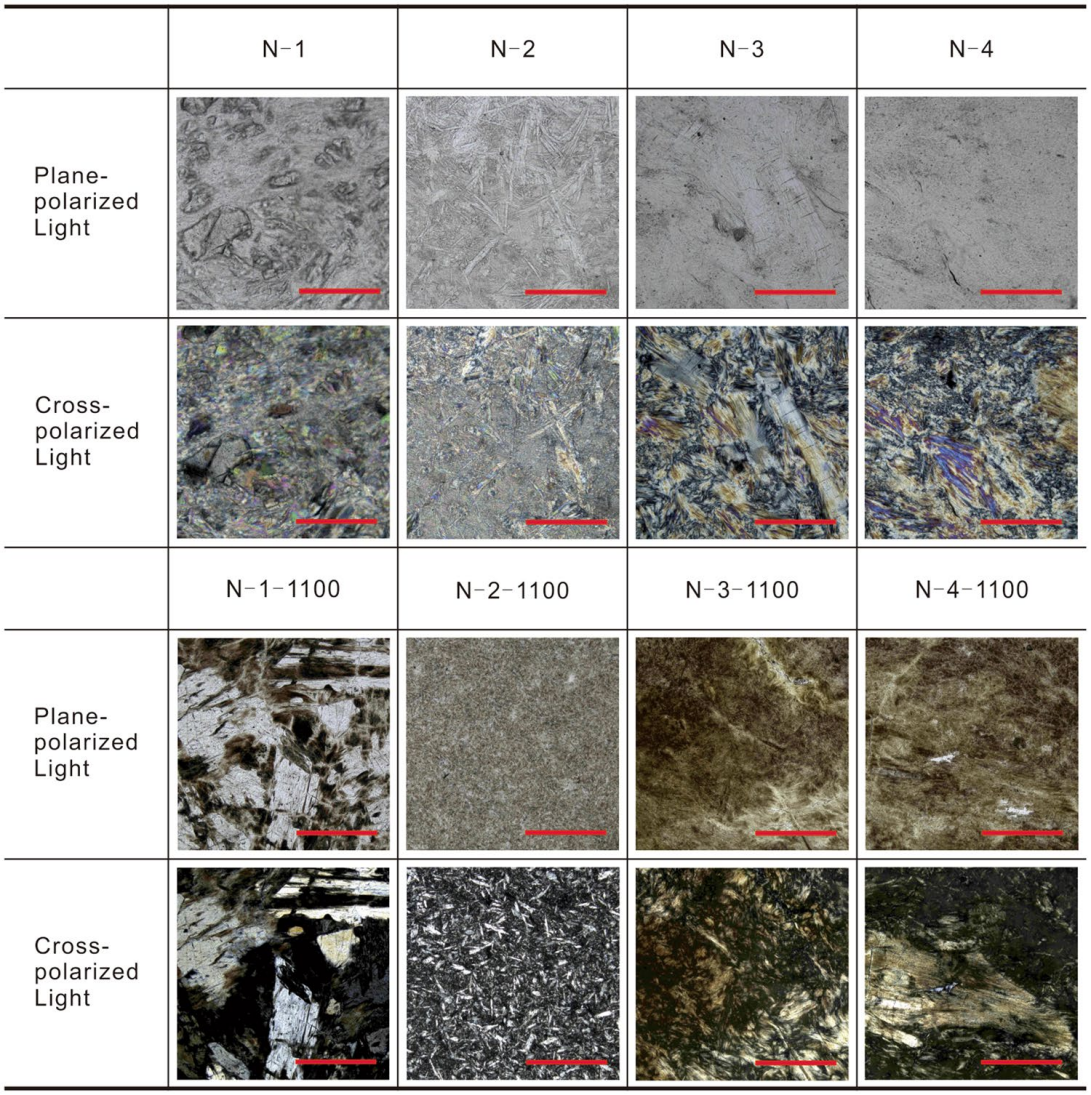
Polarized light micrographs of the heated and unheated nephrite jade samples. Unheated samples (N-1, N-2, N-3, N-4) and 1100 °C heated samples (N-1-1100, N-2-1100, N-3-1100, N-4-1100) were used to make rock-slices to be observed under a polarizing microscope. Because the crystallinity reached the best degree of this reaction when heating temperature over 1100 °C; although the transformation has already finished below 1000 °C according to the XRD data (Fig. 2). The scale bars in Fig. 3 are all 0.3 mm. N-1: tremolite is about 96%, colorless, about 0.5 × 0.15 mm, in woven fibre structure with some directionality; actinolite is about 3%, green, 0.05–0.25 mm, in woven fibre structure; epidote is about 1%, colorless to light green, 0.1–0.04 mm, in granulous and columnar structure. N-2: tremolite is about 90%, colorless to light green, about 0.4 × 0.05 mm, in woven fibre structure randomly distributed; actinolite is about 5%, green, small than 0.3 × 0.04 mm, in woven fibre structure; epidote is about 3%, colorless to light green, 0.05–0.4 mm, in schistose and scaly structure; talc is about 2%, colorless, smaller than 0.01 mm, in small scaly structure. N-3: actinolite is about 93%, light green, about 1 × 0.2 mm, in woven fibre structure; tremolite is about 6%, colorless, in woven fibre structure; magnetite is about 1%, opacity, 0.1–0.6 mm. N-4: actinolite is about 96%, light green, about 1 × 0.1 mm, in woven fibre structure; tremolite is about 3%, colorless, 0.05–0.2 mm, in small woven fibre structure; other minerals are too small to recognize.

According to Fig. 3, the heated samples show little typical optical characteristics thus the rock-slices are not useful to determine mineral species. The difference between nature and heated minerals under a polarizing microscope can help to determine whether the samples were heated before. Although this reaction reaches the best crystallinity of this reaction when the heating temperature is over 1100 °C, this crystallinity degree is still very low since the heated samples show little typical optical character.

Tremolite-actinolite in the low-iron samples N-1 and N-2 transform completely at ~1000 °C in XRD data (Fig. 2). Tremolite-actinolite in the high-iron samples N-3 and N-4 transform partially at ~900 °C with lower crystalline and completely at ~1000 °C with higher crystalline. As a result, the transform temperature become lower with the iron content increase, the transform is more concentrated with the iron content decline.

**Figure 4:**
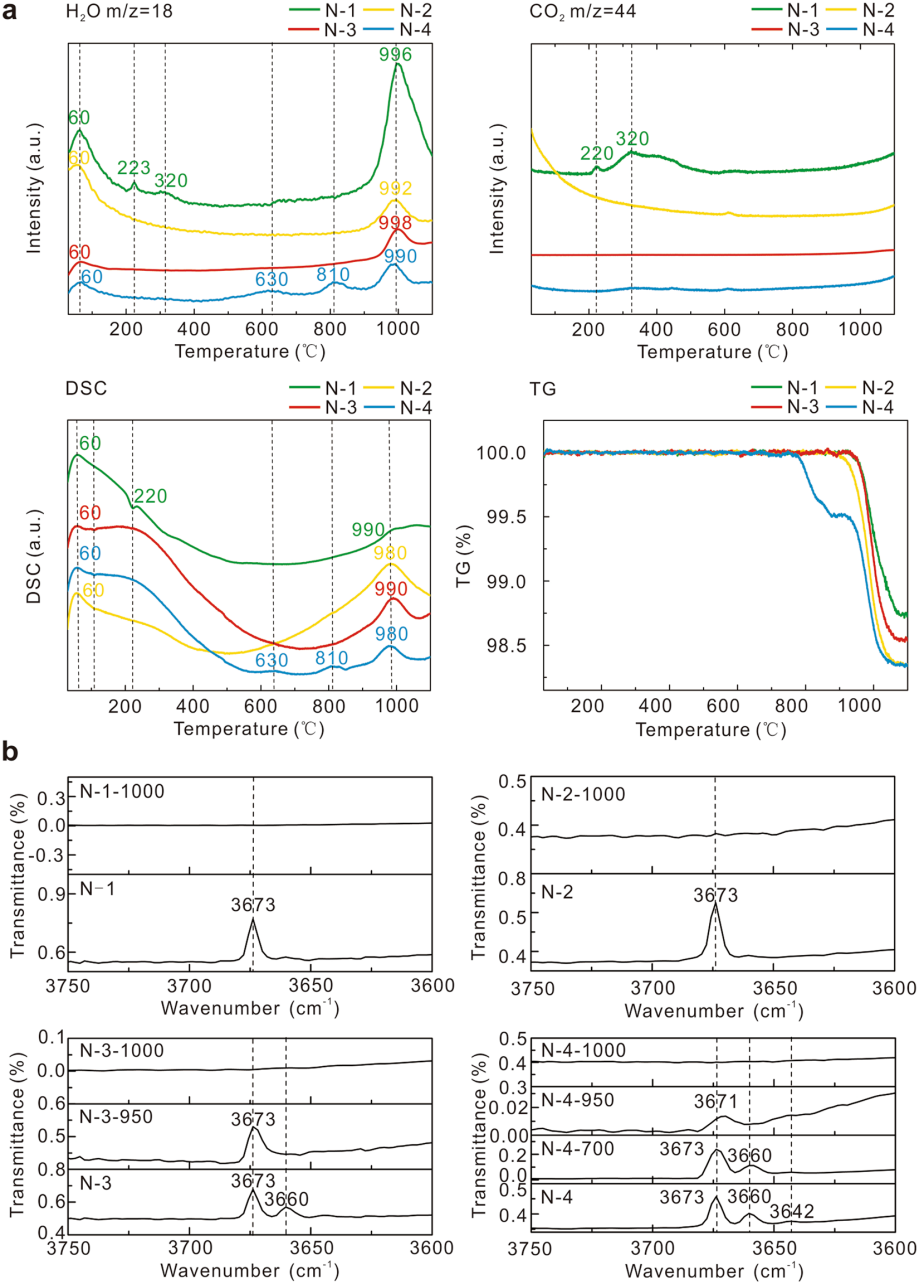
TGA and FTIR data of the nephrite jade samples. (**a**) TGA data of the nephrite jade samples. N-1 release CO_2_ and H_2_O from the carbonate masse, which is an accessory mineral, at about 220 °C and 320 °C. (**b**) FTIR data of the nephrite jade samples.

Loss of OH^−^ is the main change in the heating process according to the thermo-gravimetric analyser (TGA) data in Fig. 4a. The heat absorption and weight loss of samples synchronize with the formation of H_2_O. The H_2_O has two sources; one is vaporized absorption water which loss at about 100 °C; another is the constitution water. At the same time, the Fe^2+^ and Mg^2+^ of nephrite in M_1_ and M_3_ (the sites in nephrite crystal for metal ions) will be reallocated to new minerals.

### Constitution water

Study on the constitution water of nephrite will help narrow down the heating temperature of excavated heated jades in future research.

Nephrite has 1 to 3 peaks between 3700 cm^−1^ and 3600 cm^−1^ in the Fourier transform infrared spectroscopy (FTIR) graph. N-1 and N-2 only have a 3673 cm^−1^ peak in the 3700-3600 cm^−1^ range; N-3 has two peaks, 3673 cm^−1^ and 3660 cm^−1^; and N-4 has 3 peaks, 3673 cm^−1^, 3660 cm^−1^ and 3642 cm^−1^. The peak 3642 cm^−1^ disappears at ~700 °C, 3660 cm^−1^ disappears at ~950 °C, and 3673 cm^−1^ at ~1000 °C (Fig. 4b). The quantity, wavenumber and intensity of these peaks relate to the constitution water. In nephrite, OH^−^ bonds with one M_3_ and two M_1_. The M_1_ and M_3_ are often filled with Fe^2+^and Mg^2+^. The different combinations of Fe^2+^ and Mg^2+^ make inequivalent M_1_ and M_3_. The quantity of OH^−^ peaks increases with the rise of the iron content^23^. There are 3 compound modes of OH^−^ in nephrite, which are OH^−^(Mg, Mg, Mg), OH^−^(Mg, Mg, Fe^2+^) and OH^−^(Mg, Fe^2+^, Fe^2+^), and the infrared wavenumber falls in the same sequence. The peak of OH^−^(Mg, Mg, Mg), OH^−^(Mg, Mg, Fe^2+^) and OH^−^(Mg, Fe^2+^, Fe^2+^) are 3673 cm^−1^ 3660 cm^−1^ and 3642 cm^−1^ in FTIR, respectively. These peaks disappear in turn with the enhancement of temperature, indicating the orderly vanish of OH^−^_._

Combining FTIR data and TGA data, the loss of constitution water is an endothermic process accompanied by weight loss. The 3673 cm^−1^ peak in FTIR data corresponds to OH^−^(Mg, Mg, Mg). OH^−^(Mg, Mg, Mg) forms H_2_O at around 1000 °C accompanied with about 1% weight loss. Thus the 3673 cm^−1^ peak (OH^−^(Mg, Mg, Mg)) disappears at ~1000 °C. For the same reason, the 3660 cm^−1^ (OH^−^(Mg, Mg, Fe^2+^)) disappears at ~950 °C, the 3642 cm^−1^ (OH^−^(Mg, Fe^2+^, Fe^2+^)) disappear at ~700 °C.

### Identification method

The rules of constitution water loss based on FTIR data and XRD data can be used to determine the real heating temperature of heated jades, especially the high-iron jades (Table 2). XRD is used to determine the mineral species and FTIR can measure the kinds of OH^−^ in nephrite. The low-iron jades only have one OH^−^ peak which disappears at ~1000 °C. If tremolite-actinolite in the low-iron jades transform little into diopside-hedenbergite and still have the OH^−^ peak, it means the heating temperature is lower than 1000 °C; if the low-iron jades have no OH^−^ peak, it means the heating temperature is over 1000 °C. The temperature range can be narrowed down more for high-iron jades. Take the high-iron samples which have three peaks in the 3700-3650 cm^−1^ range in FTIR as an example. When the heating temperature is under 700 °C, the tremolite-actinolite are still one of the main minerals in the samples and the samples have three OH^−^ peaks. Between 700 °C and 900 °C, the samples lose one peak, but are still tremolite-actinolite. Between 900 °C and 950 °C, the samples have two OH^−^ peaks with some diopside-hedenbergite generated. From 950 °C to 1000 °C, the samples have only one OH^−^ peak, but still have a little tremolite-actinolite left. Over 1000 °C, there is no OH^−^ peak and all the tremolite-actinolite transforms into diopside-hedenbergite. If the high-iron samples have only two OH^−^ peaks, the estimated heating temperature region should be below 900 °C.

**Table 2:** Heating temperature judge method of nephrite jade samples.

Low-iron nephrite			High-iron nephrite		
Temperature/°C	XRD	FTIR	Temperature/°C	XRD	FTIR
<1000	Nephrite	1	<700	Nephrite	3/2
≥1000	Diopside	0	700–900	Nephrite	2
			900–950	N & D	2
			950–1000	N & D	1
			≥1000	Diopside	0

In XRD data, N means nephrite, D means diopside. The number in FTIR data means the peak number in the 3700–3650 cm^−1^.

### Structure change

The 3 silicate series in the heating process are all chain silicate mineral. Tremolite-actinolite is double-chain silicate mineral, diopside-salite and enstatite-hypersthene are single-chain silicate mineral. During the heating process, the double-chain of tremolite-actinolite breaks down; the OH^−^ in aperture and the metal ion in A site (lattice pore in nephrite crystal^13^) disappear; the metal ions at M_1_, M_2_, M_3_ and M_4_ reallocate into new minerals. The lattice constant of tremolite-actinolite is different from the new minerals which lead to the crystals fragment. After being heated, the crystal particle becomes no more than a quarter of its original size.

The tremolite-actinolite samples are all woven fibre structure before being heated, while the heated samples are schistose and scaly structure observed from rock-slices (Fig. 3). The structure of diopside-salite and enstatite-hypersthene which formed in nature are columnar. But the crystals formed in the heating process shape poor grains and tend to form schistose and scaly structure. The heated samples all become disordered after the heating process due to the fragmentation of crystals and low-degree crystallinity.

The melting temperature of diopside-salite in nature is 1391 °C. But in the heating process, the samples begin presenting the melting phenomenon from ~1200 °C and the edge parts melt at ~1300 °C. This means the crystals formed in the heating process are damageable and instable.

**Figure 5:**
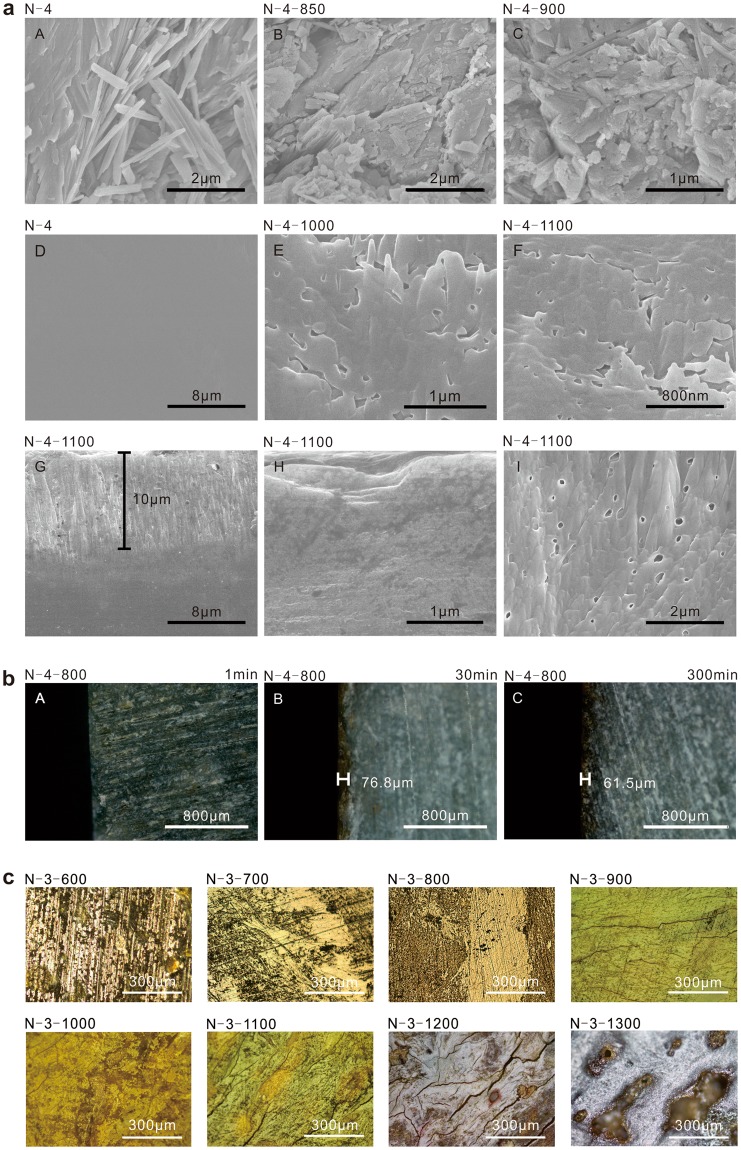
SEM images and micro photos of the heated nephrite samples. (**a**) SEM images of the heated nephrite samples. (A–C) are photos of fresh surface and (D–I) are photos of cross-sections polished by iron. The crevices and holes cross surface all belong to the samples without man-made. (**b**) Micro photos of the hierarchy of the nephrite samples heated in different holding time. (**c**) Micro photos of surfaces of the heated nephrite samples.

Holes and crevices are important features of heated samples. The holes are rear on unheated samples (Fig. 5aA). The crystals begin to turn to schistose and scaly structure at 800 °C (Fig. 5aB). The holes and crevices appear on samples since 900 °C (Fig. 5aC). The sizes of holes are general 10–50 nm and no bigger than 100 nm. The lengths of crevices are generally 200–300 nm and no longer than 320 nm, and the width is generally 50–10 nm and no wider than 140 nm. The area of holes and crevices is cover 3–4% of the whole volume and the average area is 29 nm by statistics. The holes and crevices become bigger with the increase of temperature (Fig. 5aE). The crevices on crystal surface are smaller than 10 nm (Fig. 5aF). Small holes and small crevices continuously generated when bigger holes and crevices appear. The crevices formed during the heating process on N-1 tend to concentrate around former crevices. Hence, holes and crevices tend to appear around the surface and the original flaws.

Numerous round holes are considered as an evidence to identify whether the jades were heated before. The amorphous from heating process serves as a good buffer, restraining the influence of crystal during the expending of H_2_O gas. The phenomenon of plentiful round holes is common in heated samples while rarely in unheated one. Hence, this phenomenon can serve as identification proof.

### Structure hierarchy

The samples are divided into two parts from ~800 °C, outside and inside layer. The phenomena of high-iron samples are more obvious than that of low-iron samples. Polished cross section of unheated N-4 has no hole and no crevice (Fig. 5aD). Taking N-4–1100 as an example, the structure is divided into an outside layer and an inside layer by crevices or number of holes near the line of the boundary (Fig. 5aG). The thickness of the outside layer is 90–100 μm. Its crevices have obvious orientation and their lengths are between 1 and 3 μm (Fig. 5aH). The directionality phenomenon of crevices appears in the other three samples as well. The holes in the outside layer are 0.1–1 μm, and that of the inside layer are 1-0.3 μm (Fig. 5aI). The holes and crevices of the inside layer are much smaller in size and less in number than outside layer.

The outside layer is inconsecutive. Its thickness is a random variable. The sizes are generally in the range of 10–300 μm, and up to 1000 μm. The inside layer will absence and the two outside layers will become an integral one when the thickness of the sample is small. The thicknesses of the outside layers have no noteworthy change with the rise of sample thicknesses. Consequently, the iron content and heating temperature make no remarkable difference to the thicknesses of outside layers.

Analysis the relationship between structure hierarchy and holding time. N-4 was taken to do the verification experiments. The heating rate was set as 10 °C/min. The heating temperature was set to 800 °C since the hierarchies of samples are obvious and the phase transforms little at 800 °C. The holding time was set as 1 min, 30 mins and 300 mins, respectively. The hierarchy of 1 min holding time sample is unapparent (Fig. 5bA); the hierarchy of the 30 mins holding time sample is obvious and the thickness of the outside layer is 76.8 μm (Fig. 5bB); the hierarchy of the 30 mins holding time sample is obvious and the thickness of the outside layer is 61.5 μm (Fig. 5bC). In a word, the hierarchy forms gradually when holding time is between 1 min and 30 mins; the hierarchy tends to be stable when the holding time is over 30 mins.

The difference in the ambient condition of outer and inner structures causes structure hierarchy. Outer structure contacts with air and the produced H_2_O vapor escapes out easily. When it comes to a certain depth, the H_2_O formed in inner gathers in the boundary. Then the structure breaks up at high pressure. It is necessary to explain that the boundary generally forms in the range of 500–600 °C, and the interconnected holes form in 900–1000 °C in most cases. Thus there is no channel in outer for gas at the temperature of the boundary formed. The boundary thickness is variational and always change with the structure and thickness of the samples.

### Surface structure

The surface and the prepared polishing scratches basically remained unchanged when being heated below 800 °C. The microcracks start to appear when being heated above 800 °C. The amount of cracks increases with the rise of temperature. The polishing scratches are out of shape when being heated over 1000 °C (Fig. 5c). Therefore, the polishing scratches on heated jade samples can help to study the craft and decoration when heating temperature is below 800 °C. It can also be used as reference data when heating temperature is between 800 °C to 1000 °C. But useless to study when temperature is over 1000 °C.

Glossiness relates to surface smoothness. Better glossiness comes with better surface smoothness. Glossiness changes with the transform of surface smoothness. The glossiness of heated samples basically remains unchanged when the heating temperature is below 800 °C; and gradually reduces with the increase of heating temperature and finally turns to earthy lustre.

### Transparency

The transparency of N-1 decreases between 700 °C and 900 °C, and then becomes opacity in the range of 900–1100 °C, but finally increases in 1100–1300 °C. The transparency of N-2 droops between 500 °C and 1000 °C, and then becomes opacity at 1000 °C, but finally increases in 1000–1300 °C. The transparency of N-3 falls between 400 °C and 600 °C, and becomes opacity in 600–1300 °C. The transparency of N-4 falls between 500 °C and 800 °C, and becomes opacity in 800–1300 °C. The temperature of transparency decrease falls with the increase of iron content and the decline of smoothness. The low-iron samples have three stages of transparency change, which are the decline, remaining in opacity and the increase in turn. The high-iron samples only have two stages, which are the decrease and remaining in opacity in sequence. Therefore, the content of iron may influence the change of transparency in heating process.

### Colour change

In Fig. 1, the colour change of the samples can be easily observed with the naked eye. The colour change rules of the four samples have some similarities, four samples all show some black colour between 200 to 500 °C, and begin to change to various shades of brown from ~500 °C. However, there are also some differences starting from ~500 °C. The low-iron jades turn yellowish-white or light-brownish-yellow while the high-iron jades change into yellow-brown and nigger-brown.

**Figure 6:**
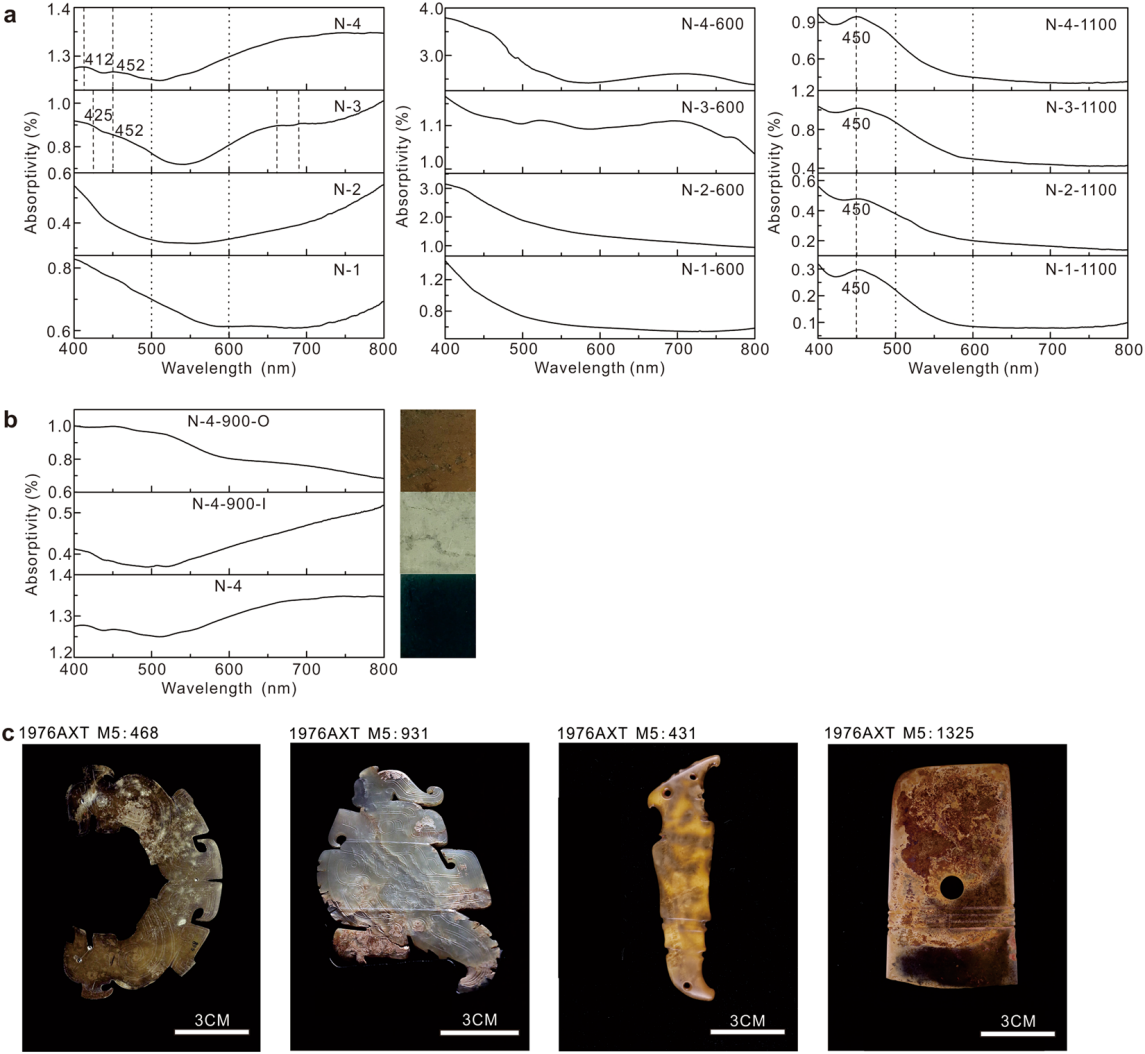
The data on colour of the heated nephrite jade samples. (**a**) UV-Vis data of the four heated jade samples. (**b**) The UV-Vis data of different colour layers of the heated jade samples. (**c**) Photos of heated ancient jades from the Yinxu Site.

The black colour variation in 200–500 °C comes from the organic matter. The organic matter in the surrounding environment decomposes in the range of 200–500 °C. These splinters spread at the surface and through the whole sample. This decomposed organic matter penetrates the structure aperture inside the samples. With the increase of temperature, the organic splinter drastically transforms into CO_2_. Meanwhile, the black colour disappears. This phenomenon can help to determine whether the jade was heated in temperature around 200 to 500 °C. For example, the black colour on 1976AXT M5:468 (Fig. 6c) unearthed from the Yinxu Site might due to the 200–500 °C heating.

Iron precipitation phenomenon is demonstrated on rock-slices of the 4 heated samples (Fig. 3). Iron precipitation in this study is the process that the iron escapes from mineral and forms iron oxides. The crystals of iron oxides which spread in crystal grain are brown. The three requirements of iron precipitation are removable iron, tunnel, and power, respectively. The free-state iron ion at A site of nephrite is the removable iron. The tunnel is the space between crystals. The power comes from heating. The iron precipitation generally starts from 500–600 °C and the colour begin to change to sap brown.

Previous research pronounced that all Fe^2+^ was oxidized to Fe^3+^ in the range of 500–600 °C^10^. Nevertheless, the samples heated in 500–600 °C displays no diagnostic absorption peak of Fe^3+^ in the ultraviolet-visible spectrophotometer (UV-Vis) (Fig. 6a). It is illustrated that Fe^2+^ in nephrite only partly oxidizes to Fe^2+^ in 500–600 °C. Dissociative Fe^2+^ is easy to be oxidized and this process leads the colour change, while the binding Fe^2+^ in the crystal structure is relatively stable. The oxidation of dissociative Fe^2+^ makes no contribution to the phase transformation.

The colour of different samples was measured by the UV-Vis spectrophotometer (Fig. 6a). The complementary colour of 600–800 nm is blue to green, and that of 400–500 nm is yellow to orange. The four samples had different UV-Vis spectrum in the original state.

The UV-Vis spectrum of unheated samples has two parts. One is 400–500 nm which form yellow hue and its colour mechanism is due to the LMCT for *O*^2−^ → *Fe*^3+^. The other is 600–800 nm which is related to green as a result of coupled pairs *Fe*^2+^*(*^*5*^*T*_2_) + *Fe*^3+^(^*6*^*A*_1_) → *Fe*^2+^(^*5*^*E)* + *Fe*^3+^(^*6*^*A*_1_) and *Fe*^2+^ → *Fe*^3+^. The bigger absorptivity, the denser of the colour is. The band at 660 nm and 690 nm of N-3 are due to *Cr*^3+^(^*4*^*A*_2_) → ^*4*^*T*_2_ and *Cr*^3+^(^*4*^*A*_2_) → ^*4*^*T*_1_ + ^*2*^*E*, respectively^12^.

With the rise of temperature, the absorption increases in 400–500 nm and declines in 600–800 nm. The 450 nm absorption peaks appear at 1000 °C and stabilize at 1100 °C. This peak comes from *Fe*^3+^(^*6*^*A*_1_) → ^*4*^*E* + ^*4*^*A*_1_(^*4*^*G*)^12^. The four samples all present the 450 nm peak, which means the Fe^3+^ becomes the main colouring element after being heated in 1000 °C. The shade of yellow-brown is combined with the colouring iron content. The colour is darker with the colouring iron content increase. On the contrary, the colour is lighter with the colouring iron content decrease. The other colouring elements have no detectable absorption, such as Cr^3+^.

N-3 and N-4 are two types of results in contrast (Fig. 1). The heating results of N-4 are from one piece of sample. The colour of samples heated from 800 °C to 1300 °C regularly changes from light to dark brown. The colour of heated samples disciplinary changes from light to dark with the rise of temperature when the colouring Fe^3+^ exists in a certain content. The heating colour on a sample can be used to determine the heating temperature of a different part. The results of N-3 are from different pieces of samples and the colour from 700 °C to 1300 °C changes irregularly. Therefore, the heating colour cannot be used to determine the heating temperature when the concentration of colouring Fe^3+^ and the structural feature is unknown. We should first determine whether the jade was heated before and then judge the iron content from the heated colour. For instance, M5:931, M5:431 and M5:1325 unearthed from the Yinxu Site (Fig. 6c) display different hue of brown and indicate the relative iron content increase in turn.

### Colour hierarchy

The colour hierarchy phenomenon of heated samples is related to the oxidation of Fe^2+^ over 500 °C. According to the UV-Vis data, the colour of the outside layer is affected by Fe^3+^; the white colour inside layer has no obvious colouring elements (Fig. 6b). The outside layer directly contacts with the air and has enough oxygen, the Fe^2+^ gradually oxidizes with the rise of temperature and the colour changes to yellowish-brown or brown simultaneously. The inside layer is out of contact with the air and lacks enough oxygen. Since the holes disappear at the outside layer and there is no tunnel to offer oxygen at 500 °C. So the inside layer lacks enough oxygen to oxidize the Fe^2+^ and the colour does not turn to yellowish-brown or brown.

The colour hierarchy of 1 min holding time sample is absent with the unchanged colour (Fig. 5bA); the hierarchy of 30 mins holding time sample is obvious with brown colour (Fig. 5bB); the hierarchy of 300 mins holding time sample is obvious with brown colour (Fig. 5bC). In a word, the Fe^2+^ is gradually oxidized to Fe^3+^ with the holding time running in 1–30 min and the colour of the outside layer gradually changes; the Fe^2+^ is almost completely oxidized to Fe^3+^ when holding time reaches ~30 mins and the colour does not change much with longer holding time. So the colour hierarchy forms gradually when holding time is between 1 min and 30 mins; the colour hierarchy tends to be stable when holding time is over 30 mins.

## Conclusions

According to the experiment, the method of determining the heated ancient nephrite jades is summarized as follows. The low-iron jades and high-iron jades are summarized separately.

### Determining the low-iron jades

First, phase transformation. The phase transform can serve as an evidence for heated jade with the heating temperature over 1000 °C (Table 2). Second, structure change. The structure of samples changes from even to loosen and forms numerous round holes. The crystal shape of samples changes from woven fibre structure to schistose and scaly structure when being heated over 800 °C (Figs 3 and 5a). Third, colour change. The samples show some black colour between 200 to 500 °C, and the color changes from greenish-white to yellowish-white or light-brownish-yellow from 500 °C (Fig. 1). Fourth, hierarchy. The outside layer is darker than inside. And the structure of the outside layer is looser than inside. Fifth, transparency and lustre. The transparency change has three stages which are decreasing to opacity, remaining in opacity and finally increasing, but the transparency deceases in general. The lustre changes from greasy lustre to earthy lustre. The transparency and lustre change can be used as secondary evidence. In conclusion, the heating temperature range can be divided into 4 stages. First, 200–500 °C, the samples show some black colour; second, 500–800 °C, the colour changes to yellowish-white or light-brownish-yellow; third, 800–1000 °C, the structure begins to change; fourth, 1000–1300 °C, the phase is completely transformed.

### Determining the high-iron jades

First, phase transformation. Combining the rule of constitution water loss with the rule of phase transformation can determine whether the samples were heated before, and can narrow the heating temperature range (Table 2). Second, structure change. The structure of samples changes from even to loosen and have numerous round holes after the heating process. The crystal shape of samples changes from woven fibre structure to schistose and scaly structure when the heating temperature is over 800 °C. (Fig. 5b). Third, colour change. The samples show some black colour between 200 to 500 °C, and the color changes from green to yellowish-brown or dark brown at a temperature starting from 500 °C (Fig. 1). Heating time and heating temperature can be contrasted when the iron contents are alike. Fourth, hierarchy. The outside layer is darker than the inside. And the structure of the outside layer is looser than inside. Fifth, transparency and lustre. The transparency change has two stages which are the decreasing and remaining in opacity. The lustre changes from greasy lustre to earthy lustre. The transparency and lustre change can be used as secondary evidence. In conclusion, the heating temperature range can be divided into 7 or 6 stages. First, 200–500 °C, the samples show some black colour; second, 500–700 °C, the colour changes to yellowish-brown or dark brown; third, 700–800 °C, the samples lose first OH^−^; fourth, 800–900 °C, the structure begins to change; fifth, 900–950 °C, the phase is partially transformed; sixth, 950–1000 °C, the samples lose second OH^−^; seventh, 1000–1300 °C, the sample lose third OH^−^, and the phase is completely transformed. The second and third stage should combine as one stage when the OH^−^ number of unheated sample is 2.

### Influence of the iron contents

The iron contents of samples greatly influence the result of the experiment. Firstly, the phase transformation temperature will be lower with the increase of iron content. Secondly, the products are influenced by the iron content. Thirdly, the colour of heated samples will be darker with the rise of iron content. Fourthly, the hierarchy of colour and structure will be more obvious with the increase of the iron content.

### Hierarchy

The hierarchy phenomenon in structure and colour is not simultaneous during the heating process. Colour hierarchy is due to the iron oxidation from ~500 °C, while structure hierarchy forms from the loss of constitution water in phase transformation starting from ~800 °C. Therefore, the thickness of the outside layer is different in most cases.

According to the result, we need to pay more attention to protect the heated ancient jades since the low crystalline, worse stability and loose structure of the heated ancient nephrite jades. First, we should determine whether the jades were heated before. Then, we should choose the best practice of protection in different on-site environment conditions.

## Data Availability

The datasets generated and/or analysed the current study are available from the corresponding author on reasonable request. All data generated or analysed during this study are included in this published article.
